# The First Reported Case of Hyperreninemic Hypoaldosteronism Due to Mucopolysaccharidosis Disorder

**DOI:** 10.7759/cureus.8487

**Published:** 2020-06-07

**Authors:** Antony Gayed, Valerie A Schott, Laura Meltzer

**Affiliations:** 1 Vascular and Interventional Radiology, Medical University of South Carolina, Charleston, USA; 2 Obstetrics and Gynecology, OhioHealth Riverside Methodist Hospital, Columbus, USA; 3 Pediatrics, Rush University Children's Hospital, Chicago, USA

**Keywords:** adrenal cortex, hypoaldosteronism, mucopolysaccharidosis iii, hyperkalemia, zona glomerulosa, heparan sulfate sulfatase

## Abstract

Mucopolysaccharidoses (MPS) are rare genetic lysosomal storage disorders caused by a deficiency of enzymes that catalyze the breakdown of glycosaminoglycans. MPS-III, also known as Sanfilippo syndrome, is caused by a deficiency of one of four enzymes that catalyze heparan sulfate proteoglycan degradation. MPS-IIIA results from a deficiency of heparan sulfatase. The natural history of MPS-IIIA is marked by progressive neurodegeneration. A nine-year-old boy with developmental delay presented with progressive three-month functional decline culminating in emergency department presentation for lethargy and immobility. Laboratory workup revealed hepatic and renal failure, coagulopathy, pancytopenia, hypernatremia, and uremia requiring emergent dialysis. He developed hyperkalemia during the second month of hospitalization, the workup of which led to a diagnosis of hyperreninemic hypoaldosteronism with normal cortisol. Blood chemistry consistent with renal hypoperfusion prompted exploration of adrenal ischemia, specifically affecting the zona glomerulosa and sparing the zona fasciculata, to explain low aldosterone with normal cortisol. Heparan sulfate (HS) normally acts as a storage site for basic fibroblast growth factor (bFGF), a paracrine stimulator of aldosterone, but accumulates in MPS-IIIA due to deficiency of heparan sulfatase. If bFGF is sequestered in HS deposits in MPS-III, then paracrine signaling is reduced, accounting for the state of hypoaldosteronism. To our knowledge, this is the first reported case of hyperreninemic hypoaldosteronism caused by an MPS disorder.

## Introduction

Mucopolysaccharidoses (MPS) are rare genetic lysosomal storage disorders occurring in approximately one in 20,000-25,0000 births [1,2]. These disorders are caused by a deficiency of enzymes that catalyze the breakdown of glycosaminoglycans, generally known as mucopolysaccharides. These partially cleaved glycosaminoglycans build up in cellular lysosomes, affecting cell functions. All types of MPS, except for type II, are inherited with an autosomal recessive pattern. MPS-II has X-linked recessive inheritance [3]. The radiographic skeletal abnormalities in MPS are described as dysostosis multiplex and include short and thick long bones, coxa valga deformity, beaked anterior inferior lower thoracic and lumbar vertebrae, and broadened ribs [4].

MPS-III or Sanfilippo syndrome is caused by a deficiency of one of four enzymes that catalyze heparan sulfate proteoglycan degradation [3,5]. Type A is the most common of the four MPS-III disorders, occurring in 0.27-1.89 of 100,000 births [1,2,5]. MPS-IIIA results from a deficiency of heparan sulfatase [3,5]. The natural history of MPS-IIIA is marked by progressive neurodegeneration. The presentation usually occurs at age two to seven years. There is developmental delay followed by progressive mental decline. The typically hyperactive patient first becomes more tractable, then sluggish, and finally bed-ridden. In this report, we report the case of a nine-year-old boy with hyperreninemic hypoaldosteronism in the setting of Sanfilippo syndrome type A. This is the first reported case of hyperreninemic hypoaldosteronism caused by an MPS disorder.

## Case presentation

**Figure 1:**
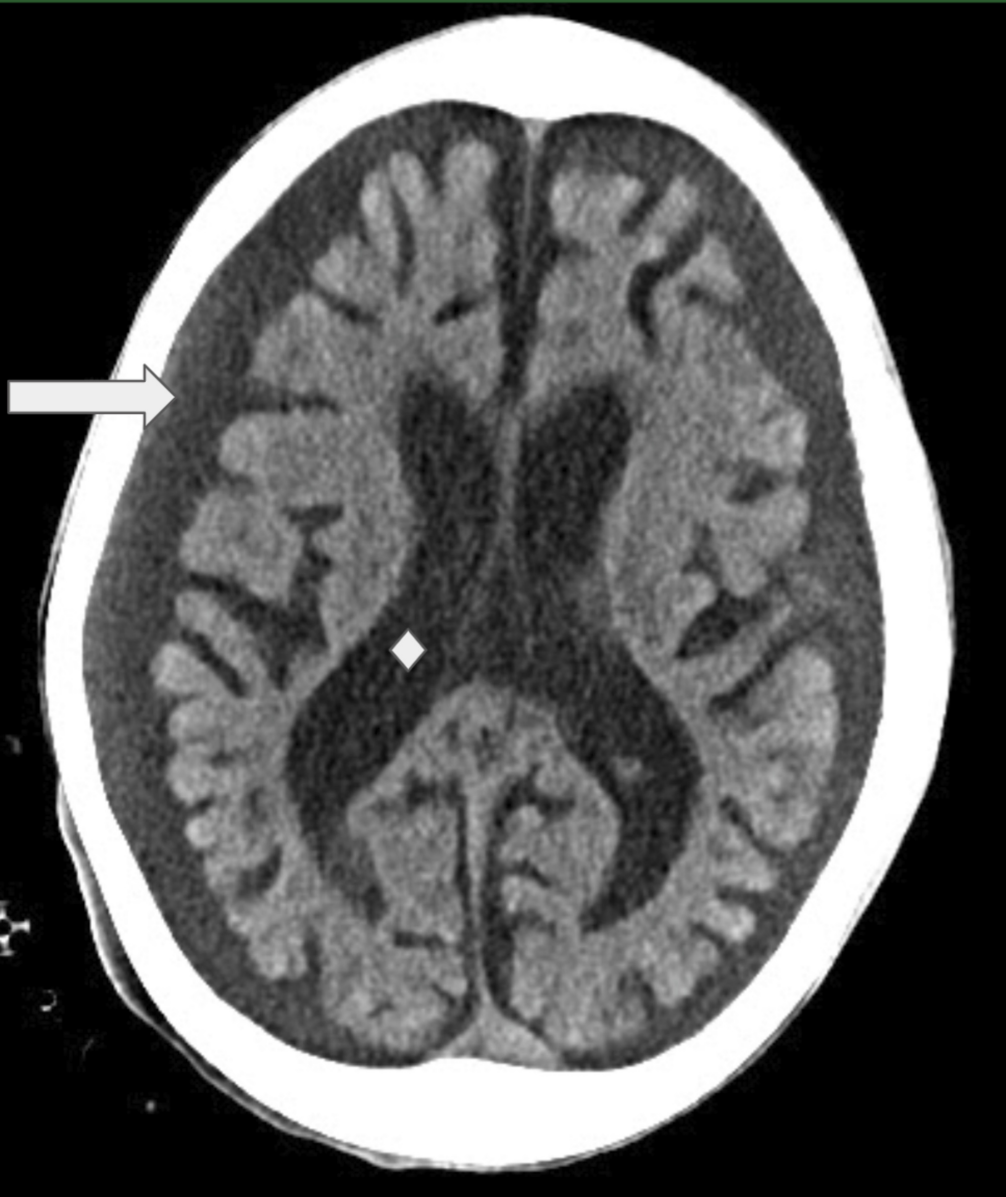
Head CT of the patient Non-contrast CT of the brain demonstrates global parenchymal volume loss as evidenced by prominent cerebrospinal fluid spaces (arrow), prominent sulcation and ex-vacuo dilatation of the lateral ventricles (diamond) CT: computed tomography

A nine-year-old boy with developmental delay presented with a progressive functional decline of three months' duration culminating in emergency department presentation for gingival bleeding, lethargy, and immobility. He had significant weight loss with malnutrition. On exam, significant vitals included a blood pressure of 99/37 mmHg and a heart rate of 58 BPM. He was lethargic with altered mental status. He had a soft S2 with grade III/VI decrescendo diastolic murmur, water hammer pulses, and contractures of all major joints. Laboratory workup revealed hepatic and renal failure, coagulopathy, pancytopenia, hypernatremia, and uremia requiring emergent dialysis. CT of the brain, obtained for the evaluation of mental status decline, demonstrated generalized atrophy (Figure 1).

**Figure 2:**
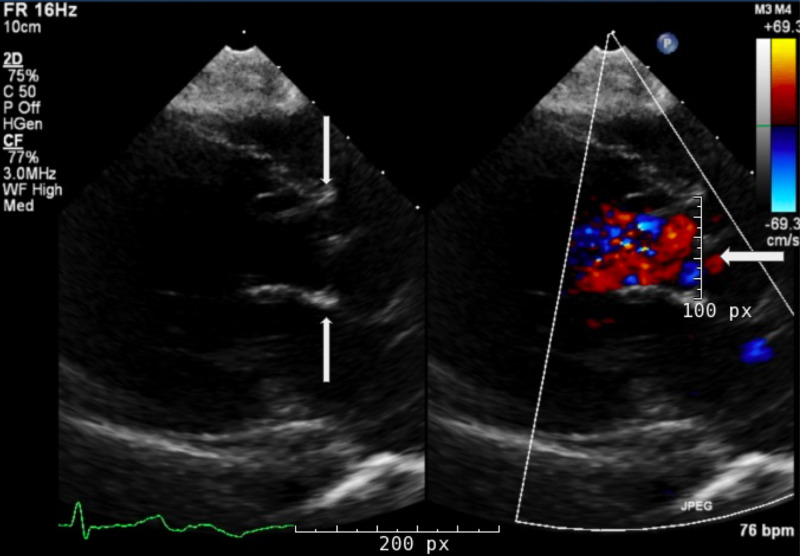
Transthoracic echocardiogram Arrows in the left image indicate the aortic valve. In the right image with color doppler, the arrow points to and indicates the direction of regurgitant flow across the aortic valve into the left ventricle

**Figure 3:**
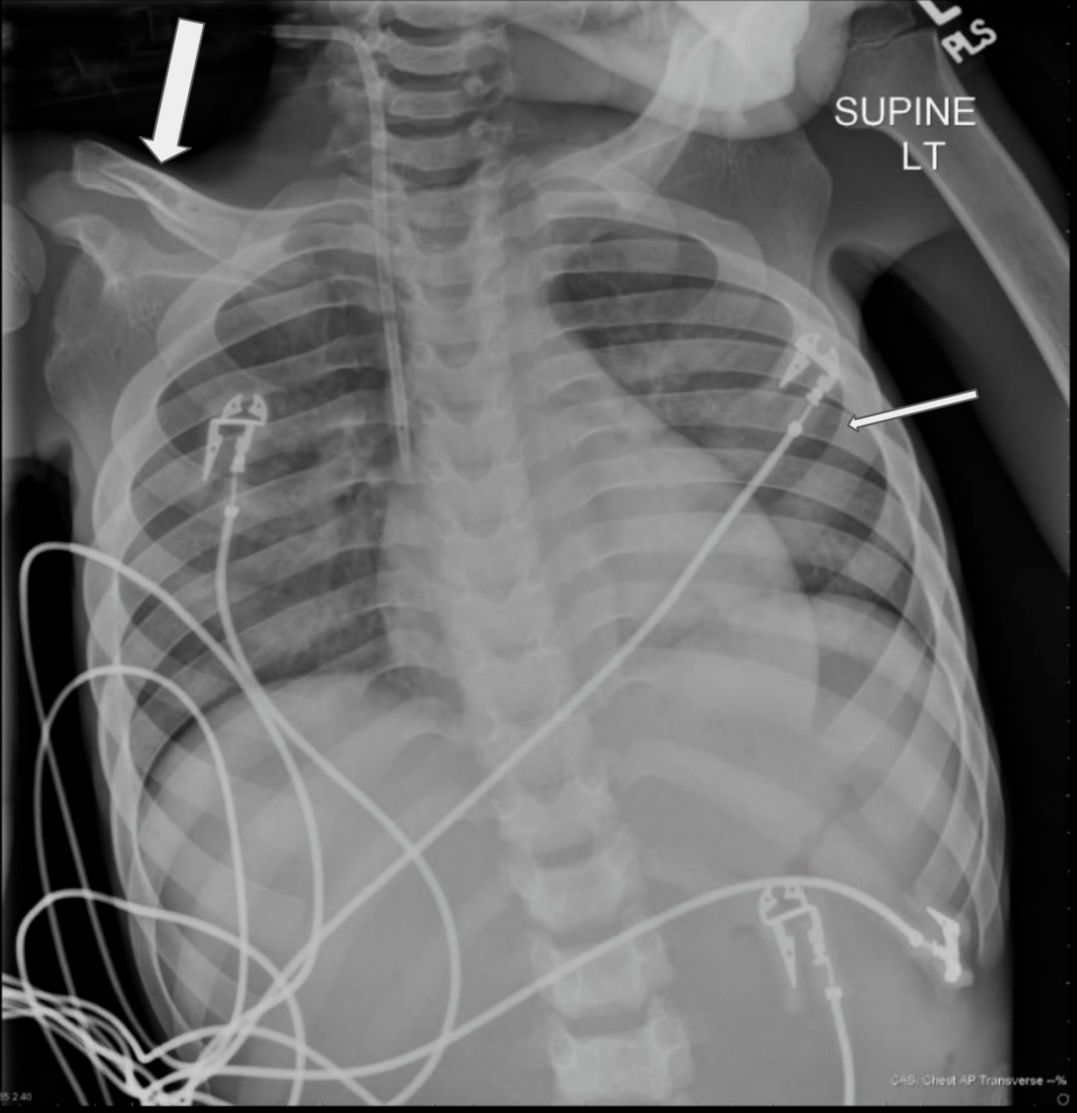
AP chest radiograph Frontal radiograph of the chest showing short and thick clavicles and broad posterior ribs. The large arrow points to the right clavicle. The clavicles are abnormally short and thick. The small arrow points to the left posterior sixth rib which is, along with the second through 11th ribs bilaterally, characteristically broad posteriorly with sparing of the most medial posterior ribs AP: anteroposterior

**Figure 4:**
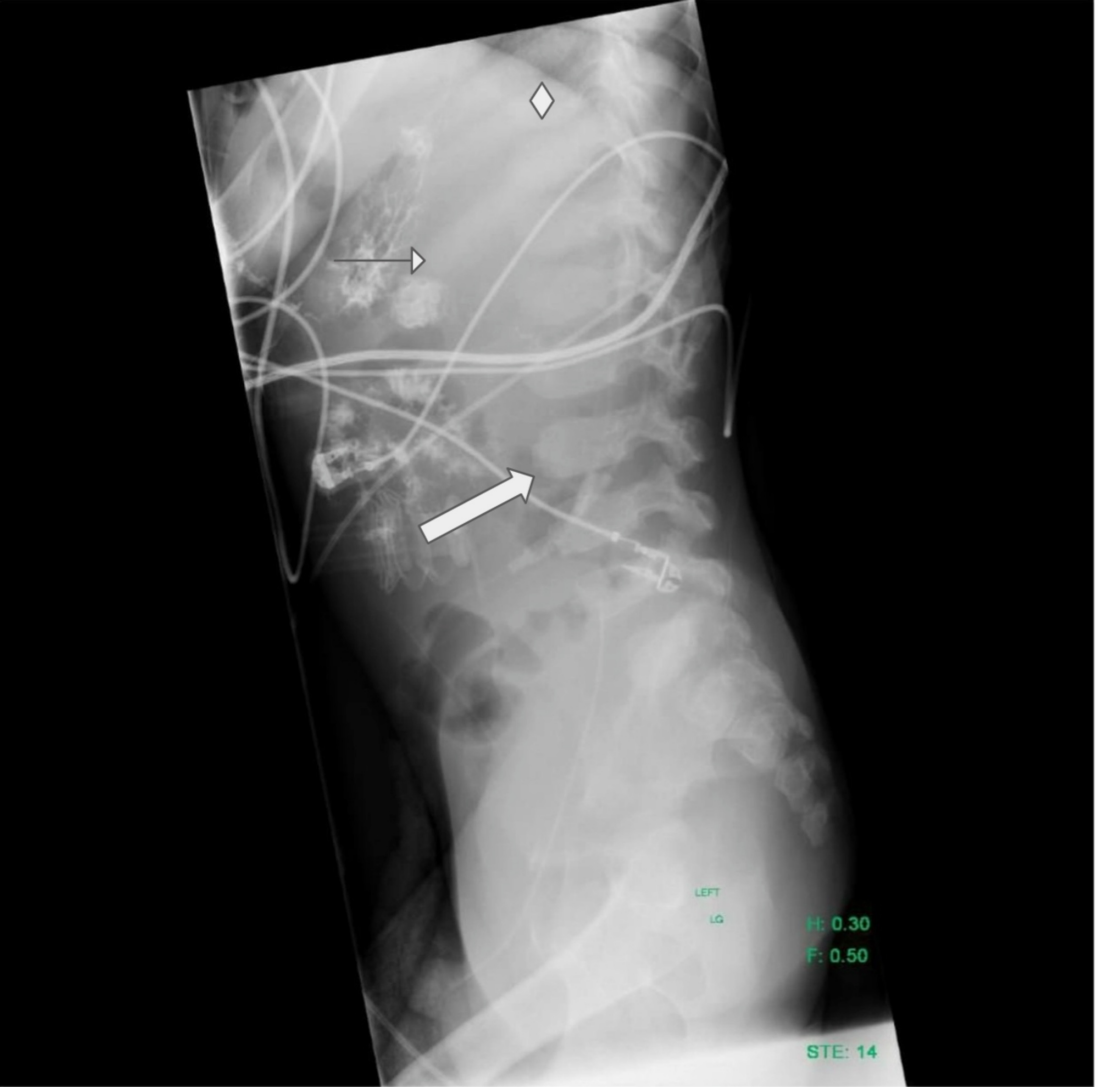
Lateral chest/abdomen radiograph The image shows beaking of the anterior aspect of the lower thoracic and lumbar vertebral bodies (large arrow) and broadening of ribs (diamond), sparing posteromedial segments (small arrow). There is intraluminal bowel opacity due to sodium polystyrene sulfonate administration

A transthoracic echocardiogram confirmed severe aortic regurgitation (Figure 2). Throughout hospitalization, his hepatic and renal failure, coagulopathy, pancytopenia, hypernatremia, and uremia improved, but he continued to require blood products. Hospital course was complicated by methicillin-sensitive* Staphylococcus aureus* bacteremia with leukocytosis and episodes of tachycardia, which were managed with antibiotics and beta-blockers, respectively. Chest and abdominal radiographs demonstrated broad posterior ribs and beaking of the vertebral bodies (Figures 3, 4); based on the imaging findings, the radiologist was the first to raise the possibility of MPS early during hospitalization. Genetic testing ultimately confirmed MPS-III, or Sanfilippo syndrome, type A. The patient developed hyperkalemia during the second month of hospitalization, and renin, aldosterone, and cortisol testing led to a diagnosis of hyperreninemic hypoaldosteronism with normal cortisol. Daily Kayexalate® (sodium polystyrene) and furosemide were required to normalize potassium levels, which eventually stabilized with hydrochlorothiazide and by mixing Kayexalate with his Ensure (Abbott Laboratories, Abbott Park, IL), a nutritional supplement, and decanting the low potassium supernatant.

## Discussion

Initially, the differential diagnosis for this patient included toxic ingestions, genetic disorders, malnutrition, neglect, infection, and malignancy due to his weight loss, altered mental status, and progressive decline in function. However, he had many features that supported the diagnosis of an MPS disorder: relative macrocephaly, developmental delay, contracted joints, coarse facies, valvulopathy, conduction abnormalities, coagulopathy, and skeletal dysostosis multiplex [3,6]. The presumptive diagnosis, initially suggested by the radiologist, was supported by acid mucopolysaccharide deposits on flow cytometry from bone marrow biopsy and confirmed with genetic testing.

Labs consistent with renal hypoperfusion prompted exploration of adrenal ischemia, specifically affecting the zona glomerulosa and sparing the zona fasciculata, to explain low aldosterone with normal cortisol. Basic fibroblast growth factor (bFGF) in animal models has been shown to act as a paracrine-stimulating agent of aldosterone downstream of angiotensin II. The receptors for bFGF are concentrated in the zona glomerulosa region of the adrenal cortex. Heparan sulfate proteoglycans of the extracellular matrix act as the storage site for bFGF and are released by heparanase or plasminogen activator proteolysis [7]. Heparan sulfate proteoglycans accumulate in lysosomes in MPS-IIIA due to the deficiency of heparan sulfatase. If bFGF is sequestered in heparan sulfate proteoglycans deposits in MPS-III, the paracrine signaling is reduced, accounting for the state of hypoaldosteronism. In pathological studies in animals with MPS III, diffuse macrovesicular vacuolation of the adrenal cortex was observed in 91% of specimens [8]. In another study, significantly higher levels of expression of genes Cy11b2 and Cy11a1 (both crucial to aldosterone production) were found in cells in the zona glomerulosa after injection of bFGF [9]. If ischemic damage occurred in the adrenals, regeneration and steroidogenesis would be delayed, specifically of aldosterone; however, aldosterone levels could make some recovery in time. This could explain the hyperkalemia including the time course correlated with low blood pressure on account of aortic regurgitation, and the gradual recovery of aldosterone requiring less potassium correction. To our knowledge, this is the first reported case of hyperreninemic hypoaldosteronism caused by an MPS disorder.

## Conclusions

In this report, we discussed a case of hyperreninemic hypoaldosteronism caused by an MPS disorder. Hyperreninemic hypoaldosteronism should be considered in the differential diagnosis of a patient with hyperkalemia and an MPS disorder.
